# Physiological Role of Bile Acids Modified by the Gut Microbiome

**DOI:** 10.3390/microorganisms10010068

**Published:** 2021-12-30

**Authors:** Yoshimitsu Kiriyama, Hiromi Nochi

**Affiliations:** 1 Kagawa School of Pharmaceutical Sciences, Tokushima Bunri University, Shido 1314-1, Sanuki 769-2193, Kagawa, Japan; nochi@kph.bunri-u.ac.jp; 2 Laboratory of Neuroendocrinology, Institute of Neuroscience, Tokushima Bunri University, Shido 1314-1, Sanuki 769-2193, Kagawa, Japan

**Keywords:** bile acids, gut microbiota, FXR, TGR5, SHP, FGF, Hippo, Mst1, Mst2, MRGPRX4, isoalloLCA

## Abstract

Bile acids (BAs) are produced from cholesterol in the liver and are termed primary BAs. Primary BAs are conjugated with glycine and taurine in the liver and then released into the intestine via the gallbladder. After the deconjugation of glycine or taurine by the gut microbiome, primary BAs are converted into secondary BAs by the gut microbiome through modifications such as dehydroxylation, oxidation, and epimerization. Most BAs in the intestine are reabsorbed and transported to the liver, where both primary and secondary BAs are conjugated with glycine or taurine and rereleased into the intestine. Thus, unconjugated primary Bas, as well as conjugated and unconjugated secondary BAs, have been modified by the gut microbiome. Some of the BAs reabsorbed from the intestine spill into the systemic circulation, where they bind to a variety of nuclear and cell-surface receptors in tissues, whereas some of the BAs are not reabsorbed and bind to receptors in the terminal ileum. BAs play crucial roles in the physiological regulation of various tissues. Furthermore, various factors, such as diet, age, and antibiotics influence BA composition. Here, we review recent findings regarding the physiological roles of BAs modified by the gut microbiome in the metabolic, immune, and nervous systems.

## 1. Introduction

**Figure 1:**
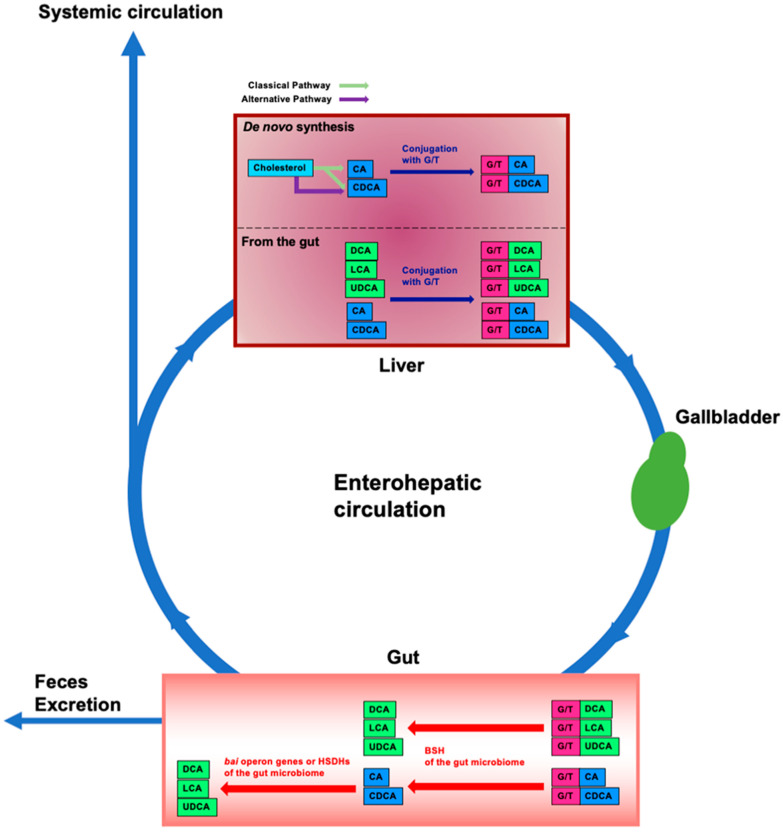
Enterohepatic circulation and modification of bile acids in the intestine by the gut microbiome. In the liver, cholesterol is converted to cholic acid (CA) and chenodeoxycholic acid (CDCA) (de novo synthesis). CA and CDCA are then conjugated with glycine or taurine (G/T). Conjugated CA and CDCA are transferred to and stored in the gallbladder; food intake stimulates their release into the intestine. Conjugated BAs are deconjugated by bile salt hydrolases (BSHs) of gut bacteria in the gut microbiome. By bile acid-inducible (*bai*) operon genes of gut bacteria, CA and CDCA are then dehydrogenated and converted to deoxycholic acid (DCA) and lithocholic acid (LCA), respectively. CDCA is also converted to ursodeoxycholic acid (UDCA) by dehydrogenation and epimerization by hydroxysteroid dehydrogenases (HSDHs) of gut bacteria. Approximately 95% of BAs in the intestine are reabsorbed and transported to the liver, and the remainder is excreted in the feces. This circulation between the gut and the liver is termed enterohepatic circulation. Some of the BAs reabsorbed from the intestine spill into the systemic circulation. Unconjugated BAs from the gut are conjugated with G/T in the liver. Blue boxes represent primary BAs. Green boxes represent secondary BAs. Pink boxes represent glycine or taurine (G/T).

Bile acids (BAs) are amphipathic steroid acids produced from cholesterol in the liver, and de novo synthesized bile acids in the liver are termed primary BAs. Primary BAs are conjugated with glycine and taurine in the liver and are then stored in the gallbladder. BAs are released from the gallbladder into the small intestine via food intake to facilitate the digestion and absorption of lipids and lipophilic vitamins by forming micelles in the small intestine. While most of the BAs are absorbed in the intestine, there is a significant deconjugation of conjugated BAs prior to reabsorption in the terminal ileum. BAs that are not absorbed in the intestine are converted to secondary BAs by the gut microbiome and these secondary BAs are then absorbed into the colon via passive diffusion [1,2]. After BAs reabsorbed from the gut are transported to the liver through the portal vein, they are conjugated with glycine and taurine in the liver and are secreted into the bile again for enterohepatic circulation (Figure 1).

As BAs are amphipathic, they can affect cell-surface and intracellular membranes, including those of mitochondria and the endoplasmic reticulum (ER). Recent studies suggest that BAs are also hormones or signaling molecules because they can bind to several nuclear and cell-surface receptors, including farnesoid X receptor (FXR) and Takeda G protein receptor 5 (TGR5)—also known as G protein-coupled bile acid receptor 1 (GPBAR1) [3,4]. By activating these receptors, BAs can regulate BA, glucose, and lipid metabolism in various tissues, including the liver, pancreas, and both brown and white adipose tissue [5]. BAs also affect the immune system [6]. Furthermore, because more than 20 BAs have been detected in the brain of humans and rodents, BAs can affect the nervous system [7,8]. In this review, we address the current knowledge on the physiological role of BAs modified by the gut microbiome in the metabolic, immune, and nervous systems.

## 2. BA Production and Modification

### 2.1. BA Production in the Liver

BAs are principally produced in the liver via the classical or alternative pathways [4,9]. Over sixteen enzymes participate in the biosynthesis of BA from cholesterol in the liver [10]. The first step of the classical pathway is the conversion of cholesterol to 7α-hydroxycholesterol by cytochrome P450 (CYP) 7A1 (CYP7A1). CYP7A1 converts cholesterol to 7α-hydroxycholesterol, which is then converted to 7α-hydroxy-4-cholesten-3-one. CYP8B1 is involved in the generation of cholic acid (CA) from 7α-hydroxy-4-cholesten-3-one, which is also converted to chenodeoxycholic acid (CDCA) by CYP27A1. The first step of the alternative pathway is the conversion of cholesterol to (25R)-26-hydroxycholesterol by CYP27A1 [11]. (25R)-26-hydroxycholesterol is then converted to CDCA by CYP7B1. CA and CDCA are then conjugated with glycine or taurine by bile acid-CoA: amino acid N-acyltransferase (BAAT) [12]. The transportation of BAs from hepatocytes into the bile canaliculi is mediated by the bile salt export pump (BSEP) and multidrug resistance-associated protein 2 (MRP2). BSEP is the predominant transporter of BAs from hepatocytes to the bile canaliculi [3,13]. BAs are preserved in the gallbladder until food intake stimulates their release into the small intestine.

### 2.2. BA Modification by the Gut Microbiome

BAs are deconjugated, dehydroxylated, dehydrogenated, and epimerized by the gut microbiome (Figure 1). Conjugated BAs from the liver are mainly deconjugated in the small intestine by bile salt hydrolases (BSHs), which are found in gut bacteria [14], such as *Lactobacillus* spp. [15,16], *Bifidobacterium* spp. [16], *Enterococcus* spp. [17], *Clostridium* spp. [18,19], and *Bacteroides* spp. [20,21]. Most of these gut bacteria exist in the ileum and colon [22]. After glycine or taurine deconjugation, CA and CDCA are converted to deoxycholic acid (DCA) and lithocholic acid (LCA), respectively, by removing the 7α-hydroxy group. Only a few bacteria have been identified that are capable of 7α-dehydroxylation; these bacteria belong to *Clostridium* spp. [22]. The 7α-dehydroxylation of CA or CDCA is performed by several proteins encoded by the bile acid-inducible (*bai*) operon. BaiB ligates CoA to the unconjugated BA. BaiA oxidizes the 3α-hydroxyl group. BaiCD catalyzes the formation of the C_4_=C_5_ bond. BaiF hydrolyzes CoA. BaiE catalyzes the 7α-dehydration, which is a rate-limiting step. BaiH catalyzes the removal of the C6=C7 bond and BaiCD catalyzes the removal of the C4=C5 bond. BaiA converts the 3-oxo-intermediate to a secondary BA [2,23]. Furthermore, 7α-hydroxysteroid dehydrogenase (7α-HSDH) and 7β-HSDH dehydrogenate epimerize and convert CDCA to ursodeoxycholic acid (UDCA) [2,24,25]. HDSHs convert DCA and LCA to iso-DCA and iso-LCA, respectively [26]. Additionally, *Eggerthella lenta* strains also possess 7α-HSDH and produce 7-Oxo-DCA from CA [27]. 7-Oxo-DCA is converted to DCA by *Clostridium* spp. [23,28]. *Eggerthella lenta* also has 3α- and one 3β-HSDH for converting DCA to isoDCA. Since IsoDCA is less toxic to bacteria than DCA, the conversion of DCA to isoDCA leads to the growth of bacteria in the gut [29]. Additionally, a recent study has demonstrated that 25 strains in the gut microbiota conjugate glycine to DCA, CDCA, or CA, and 28 strains in the gut microbiota conjugate other amino acids, namely, alanine, arginine, aspartate, asparagine, glutamate, glutamine, histidine, lysine, methionine, serine, tryptophan, and valine [30]. Furthermore, CA conjugated with phenylalanine and tyrosine by *Clostridium bolteae* strains significantly activates FXR [31].

### 2.3. BA Circulation

Approximately 95% of BAs are reabsorbed from the gut, and the remainder is excreted in the feces [32]. Reabsorption primarily occurs in the small intestine and also at a low rate in the colon. Conjugated BAs are transported into the enterocytes via apical sodium-dependent bile acid transporters (ASBTs) in the apical brush border [1,33,34,35,36]. Ileal bile acid-binding protein (I-BABP) mediates the intracellular transport of BAs from the apical to the basolateral side of enterocytes [37]. Organic solute transporter (OST) α and β in the basolateral membrane of enterocytes transport BAs from the enterocytes into the portal circulation [38,39,40]. BAs are then translocated from the portal circulation to the liver via Na^+^-dependent and Na^+^-independent transporters. Na^+^-taurocholate co-transporting polypeptide (NTCP) transports BAs into the hepatocytes in a Na^+^-dependent manner [41,42,43]. Organic anion-transporting polypeptide 1B1 (OATP1B1) and OATP1B3 transport BAs into the hepatocytes in a Na^+^-independent manner [44]. OATP1B1 and OATP1B3 transport conjugated BAs more efficiently than unconjugated BAs [45]. The reabsorbed unconjugated BAs transported to the liver are also conjugated and released along with newly synthesized BAs (Figure 1). Therefore, unconjugated primary and secondary BAs and G/T conjugated secondary BAs are modified by the gut microbiome. This recycling process between the gut and the liver is termed enterohepatic circulation. However, some of the BAs reabsorbed from the intestine spill into the systemic circulation (Figure 1). The concentration of BAs in the portal vein is ~80 μM and the concentration of BAs in the systemic circulation is ~2–10 μM [46]. Thus, because BAs in the systemic circulation can affect various tissues, BAs play physiological roles.

### 2.4. Factors That Influence Bile Acid Composition

Specific diet components influence BA composition. High- and low-fat diets are associated with a decrease in the synthesis of primary BAs [47]. In feces, a high-fat diet increases DCA, LCA, and CDCA [48,49]. This increase in secondary bile acids, DCA, and LCA is related to cancer development [50]. Additionally, proteins affect the BA composition. High-protein diets increase the plasma concentration of DCA, CDCA, and CA [51]. Soybean protein isolate increases secondary BAs, the pool of BAs, and the secondary to primary BA ratio in the cecum and feces [52]. Milk protein intake increases total and primary BAs in the serum of obese, but not overweight, subjects [53]. As for carbohydrates, a whole grain diet increases the plasma concentrations of taurolithocholic acid (TLCA), taurocholic acid, glycocholic acid, taurochenodeoxycholic acid, and glycochenodeoxycholic acid compared with a refined grain diet [54].

The plasma concentrations of most BAs decrease with age in men but not in women. A decrease in CDCA, GCDCA, TCDCA, and GUDCA is significantly related to age in men. The concentrations of DCA, GCA, UDCA, GDCA, and TDCA decrease with age in both men and women. Additionally, the concentration of DCA, GCA, and UDCA is significantly different between sexes [55]. Furthermore, the concentration of isoLCA, 3-oxoLCA, alloLCA, 3-oxoalloLCA, and isoalloLCA in the feces of centenarians is higher than in that of other age subjects [56].

Antibiotic treatment changes the gut microbiota by reducing specific species and modifying the bile acid composition [57,58]. Vancomycin and gentamicin reduce the total bacteria and intestinal microbiota diversity, leading to a change in the BA composition. These antibiotic treatments increase levels of conjugated primary BAs and decrease unconjugated primary and secondary BAs [59].

## 3. Physiological Role of BAs Modified by the Gut Microbiome

FXR and TGR5 are the most widely studied BA receptors [60]. BAs also regulate other nuclear and cell-surface receptors [4,61,62,63]. Nuclear receptors regulated by BAs include the constitutive androstane receptor (CAR), pregnane X receptor (PXR), vitamin D receptor (VDR), liver X receptor (LXR), and glucocorticoid receptor (GR). Cell-surface receptors regulated by BAs include sphingosine-1-phosphate receptor 2 (S1PR2), Mas-related G protein-coupled receptor X4 (MRGPRX4), and the M2 and M3 muscarinic receptors. Thus, BAs play a variety of physiological roles by regulating these receptors or other mechanisms.

### 3.1. The Role of Modified BAs in the Metabolic System

The main BA receptors are FXR and TGR5. BA binding to FXR and TGR5 regulates BA, glucose, and lipid levels. CDCA and DCA have a high affinity for FXR [64,65,66,67] and conjugated DCA acts as a ligand for FXR with NTCP—a BA transporter expressed in the cell [64].

FXR regulates the expression of various genes by binding to specific DNA sequences in the genome, either as a monomer or as a heterodimer with the retinoid X receptor (RXR). These specific DNA sequences are an inverted repeat of AGGTCA separated by one nucleotide (IR1), IR0, an everted repeat of AGGTCA separated by two or eight nucleotides (ER2 or 8), and a direct repeat of AGGTCA separated by one, four, or five nucleotides (DR1, 4, or 5) [68,69,70,71,72]. FXR also regulates epigenetic modifications. The activation of FXR recruits steroid receptor coactivator 1 (SRC1), which has histone acetyltransferase (HAT) activity in its C-terminal region, leading to the acetylation of histone H3 at the promoter of the *glucagon-like peptide 1 (GLP-1) receptor* [73,74,75]. In addition, FXR induces the expression of microRNA-29a, which downregulates DNA methyltransferases [76] and histone deacetylase (HDAC) 4 [77]. It has been shown that the percentage of common DNA binding sites of FXR between the liver and intestine is only 11% [69]. This suggests that the pattern of gene expression regulated by FXR is tissue-specific. Furthermore, FXR induces the expression of TGR5 [78].

TGR5 is a member of the G-protein-coupled receptor (GPCR) superfamily and couples to Gα_s_. Activated TGR5 leads to the activation of adenylyl cyclase, an increase in cyclic AMP (cAMP) concentration, and the activation of protein kinase A (PKA) and exchange proteins directly activated by cAMP (EPAC) [79]. LCA, DCA, CDCA, CA, and conjugated forms of these BAs activate TGR5. LCA and DCA, which are secondary BAs, and their conjugated forms were reported as the most effective TGR5 activators [80,81,82]. Additionally, UDCA is a weak activator of TGR5 [81,83].

#### 3.1.1. BA Metabolism

**Figure 2:**
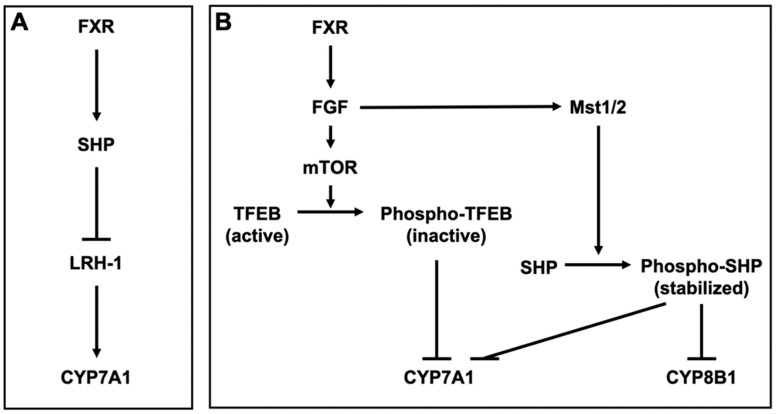
Bile acid metabolism by FXR (**A**) Farnesoid X receptor (FXR) induces small heterodimer partner (SHP) and SHP inhibits liver receptor homolog-1 (LRH-1), which induces cytochrome P450 (CYP) 7A1 in the hepatocyte. (**B**) FXR induces fibroblast growth factor (*FGF*) 15/19 (mouse FGF15 and human FGF19) and FGF15/19 in the enterocytes. In the hepatocyte, FGF15/19 activates FGF receptor 4 (FGFR4) and its coreceptor β-Klotho (βKL), leading to the phosphorylation of transcription factor EB (TFEB) by the mechanistic target of rapamycin (mTOR). TFEB induces CYP7A1 and is inactivated by the phosphorylation. The binding of FGF15 to FGFR4 activates mammalian sterile 20-like kinases 1 (Mst1) and Mst2, homologs of the Hippo kinase in Drosophila. MST1 and 2 phosphorylate and stabilize SHP, which represses CYP7A1 and CYP8B1.

FXR inhibits BA synthesis by inducing small heterodimer partner (SHP), a nuclear receptor that lacks a ligand-binding domain [84]. SHP interacts with and represses nuclear receptors and transcription factors, including PXR, CAR, LXR, GR, and FXR [85,86]. SHP also inhibits the activation of liver receptor homolog-1 (LRH-1), which induces CYP7A1, the key enzyme responsible for BA synthesis from cholesterol [87] (Figure 2A). Furthermore, FXR also inhibits the expression of CYP7A1 by inducing fibroblast growth factor (FGF) 15/19 (mouse FGF15 and human FGF19) and β-Klotho (βKL). βKL forms binary complexes with FGF receptor 4 (FGFR4) to function as a coreceptor of FGFR4 for FGF15/19 [88]. Transcription factor EB (TFEB) is activated by dephosphorylation and induces CYP7A1. After activating FGFR4/βKL by FGF15/19, the mechanistic target of rapamycin phosphorylates and inactivates TFEB, leading to CYP7A1 repression [89]. FGF15/19 also stabilizes SHP, which represses CYP7A1 and CYP8B1. CYP8B1 is a key enzyme for the synthesis of CA. The binding of FGF15 to FGFR4 activates the Hippo signaling pathway. In the Hippo signaling pathway, SHP is phosphorylated and stabilized by mammalian sterile 20-like kinase 1 (Mst1) and Mst2, homologs of the Hippo kinase in Drosophila [90] (Figure 2B). FXR also regulates BA transporters. FXR represses NTCP in the liver, leading to a reduction of the hepatic uptake of Bas [91]. On the other hand, FXR enhances BA efflux into the liver by inducing BSEP and OSTα/β [92,93]. Overall, FXR reduces excess Bas in the liver. Although TGR5 is weakly expressed in the liver, TGR5-knockout mice exhibit a decrease in total BA pool size [94]. Additionally, TGR5-knockout mice exhibit reduced levels of CYP7B1 and increased levels of secondary and hydrophobic Bas [95,96,97]. PXR is activated by LCA [98,99] and PXR regulates the expression of CYP3A and CYP2B, which convert LCA to hyocholic acid (HCA) and UDCA [100,101,102]. In addition, PXR indirectly suppresses the expression of CYP7A1 by inhibiting the activation of hepatocyte nuclear factor 4α, which upregulates the expression of CYP7A1 [103,104]. VDR is also activated by LCA and its metabolite, 3-keto LCA [105,106], and LCA induces the expression of CYP3A [106,107,108]. LXRα is weakly activated by hyodeoxycholic acid (HDCA) [109]. The BA pool size and its excretion are decreased in LXRα-knockout mice. LXRα-knockout mice also exhibit reduced CYP7A expression [110], but LXRβ-knockout mice do not [111].

#### 3.1.2. Glucose Metabolism

Glucose and insulin upregulate and downregulate FXR expression, respectively, and FXR is associated with glucose metabolism [112,113]. The association between FXR and glucose metabolism is differently affected by the fed and fasting states. In the fed state, treatment of wild-type mice with the FXR ligand decreases the expression of phosphoenolpyruvate carboxykinase (PEPCK) and glucose 6-phosphatase (G6Pase). PEPCK and G6Pase are essential for the upregulation of gluconeogenesis. In contrast, treatment of wild-type mice in the fasting state with FXR ligand increases the expression of PEPCK and G6Pase. FXR-knockout mice exhibit decreased blood glucose levels, and PEPCK and G6Pase expression are downregulated. The effects of FXR with the FXR ligand are mediated by GR [114]. The treatment of wild-type mice with CA reduces blood glucose levels and PEPCK and GP6ase expression. Furthermore, the treatment of SHP-knockout mice with CA increases blood glucose levels. Thus, the blood glucose-lowering effect of FXR is, at least in part, mediated by SHP [115]. FXR also inhibits PEPCK and G6Pase expression by inducing FGF15/19. FGF15/19 inactivates the cAMP regulatory element-binding protein (CREB), the transcription factor that induces peroxisome proliferator-activated receptor (PPAR)-γ coactivator-1α (PGC-1α) [116]. PGC-1α is a coactivator that induces the expression of genes involved in metabolic pathways and mitochondria by interacting with a variety of nuclear receptors [117,118]. FXR interacts with the carbohydrate responsive element binding protein (ChREBP) and suppresses the activity of ChREBP. ChREBP is a transcription factor that regulates the expression of genes controlling glycolysis and lipogenesis. ChREBP upregulates the expression of L-pyruvate kinase (L-PK), a key enzyme for glycolysis [119]. Activated FXR releases ChREBP and its coactivators (p300 and CREB-binding protein) from the L-PK promoter region and inhibits L-PK expression [120]. FXR and CDCA induce glucose transporter 4 expression in differentiated 3T3-L1 adipocytes and the hepatic cell line HepG2 [121]. FXR increases glucose-stimulated insulin secretion and expression by inducing the KLF11 transcription factor [113]. Although CDCA increases the glucose-stimulated secretion of insulin from wild-type mice, FXR-knockout mice are unaffected [122].

**Figure 3:**
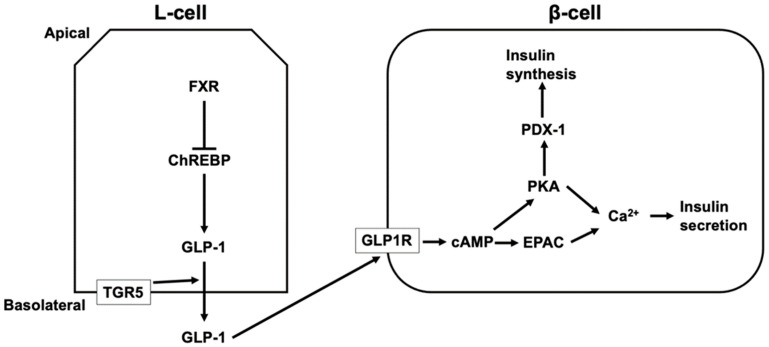
The regulation of insulin secretion and synthesis via TGR5 and FXR. TGR5 located in the basolateral membrane of L-cells mediates glucagon-like peptide 1 (GLP-1) secretion from L-cells, and secreted GLP-1 from the intestine reaches pancreatic β-cells via the systemic circulation. The binding of GLP-1 to GLP-1 receptor (GLP1R) increases cyclic AMP (cAMP) levels, leading to the activation of protein kinase A (PKA) and exchange proteins directly activated by cAMP (EPAC), which induces an increase in intracellular Ca^2+^ and insulin secretion. In addition, the activation of PKA by GLP-1 leads to the activation of pancreatic duodenal homeobox-1 protein (PDX-1), which binds to the promoter of the gene encoding insulin and induces the synthesis of insulin. FXR suppresses GLP-1 production in L-cells by inhibiting the activity of a carbohydrate responsive element binding protein (ChREBP) that induces the expression and secretion of GLP-1 in L-cells.

TGR5 activation increases the intestinal level of GLP-1 [123,124,125,126,127]. LCA, taurodeoxycholic acid (TDCA), and TLCA increases GLP-1 secretion via TGR5 activation [123,125]. GLP-1 release is mediated by TGR5 located in the basolateral membrane of L-cells [125]. Most active GLP-1 is degraded in the intestine and liver and 10–15% of secreted GLP-1 from the intestine reaches the pancreas via the systemic circulation [128]. GLP-1 induces the release and synthesis of insulin in β-cells in the pancreatic islets. The binding of GLP-1 to GLP-1 receptor activates adenylate cyclase, leading to increased cAMP levels. PKA and EPAC are then activated, which induces an increase in intracellular Ca^2+^ from the ER and extracellular sources via voltage-dependent Ca^2+^ channels [128,129]. Additionally, the activation of PKA by GLP-1 leads to the activation of pancreatic duodenal homeobox-1 protein (PDX-1), which binds to the promoter of the gene encoding insulin and induces the synthesis of insulin [128,130] (Figure 3). In contrast to TGR5, FXR suppresses GLP-1 production in L-cells by inhibiting the activity of ChREBP, which induces the expression and secretion of GLP-1 in L-cells [120,131]. Furthermore, the level of serum HCA is lower in diabetics and people with a high body mass index [132,133,134]. HCA induces GLP-1 by activating TGR5 and inhibiting FXR, and TGR5 activation increases serum insulin and decreases blood glucose levels [132].

#### 3.1.3. Lipid Metabolism

Dietary lipids (triglycerides [TGs], cholesterol, and phospholipids) are absorbed and incorporated into chylomicrons in the intestine. Chylomicrons are then converted to very low-density lipoprotein (VLDL) in the liver. VLDL transports lipids from the liver to the systemic circulation. VLDL is then converted to HDL and returned to the liver [135]. In the process of lipogenesis, acetyl-CoA carboxylase (ACC) converts acetyl-CoA to malonyl-CoA, and fatty acid synthase (FAS) converts malonyl-CoA to palmitate. Stearoyl-CoA desaturase (SCD) mediates the conversion of saturated fatty acids (FAs) to monounsaturated FAs, and the newly-produced FAs are then stored as TGs. Sterol regulatory element-binding protein 1C (SREBP1C) is a transcription factor that induces lipogenic genes, including FAS, ACC, and SCD [136,137]. FXR-knockout mice exhibit an increase in SREBP1C, SCD-1, and FAS and also exhibit increased levels of serum and liver TGs, serum cholesterols, and serum free FAs (FFAs) [115]. Additionally, CA feeding decreases the serum levels of TGs, cholesterol, and FFAs in mice [115,138]. CA and CDCA decrease the expression of FAS, ACC, SCD, and SREBP1C [138]. SHP induced by FXR suppresses lipogenesis by suppressing lipogenic genes via the inhibition of LXR, which induces SREBP1C expression [138,139]. Furthermore, FXR induces the expression of carboxylesterase 1 (CES1), which reduces hepatic TGs by inducing TG hydrolysis to release FFAs. Released FFAs activate PPARα, leading to the enhancement of FA oxidation [140,141].

### 3.2. Role of Modified BAs in the Immune System

The liver in FXR-knockout mice exhibits an elevated expression of pro-inflammatory genes, such as interleukin-1 (IL-1), IL-6, interferon-γ (IFN-γ), inducible nitric oxide synthase, and cyclooxygenase-2. FXR activation inhibits the expression of these genes by inhibiting the activity of nuclear factor-κB (NF-κB), and also has an anti-inflammatory function [142]. Moreover, CDCA inhibits the production of IL-1, IL-6, and tumor necrosis factor α (TNFα) by human monocytes [143]. FXR activation by agonists leads to conjugation with small ubiquitin-like modifier (SUMO) 2. SUMOylated FXR interacts with NF-κB and nuclear receptor corepressor (NCOR) 1 or NCOR2, also called silencing mediator of retinoic acid and thyroid hormone receptor. NCORs are transcriptional corepressors that repress pro-inflammatory gene expression in the liver [144,145,146,147]. FXR also upregulates SHP. SHP overexpression inhibits NF-κB activation and leads to the downregulation of pro-inflammatory genes in the liver. In contrast, SHP deletion activates NF-κB. Thus, FXR can suppress NF-κB activation by inducing SHP [148]. The inhibitory effect of SHP on NF-κB is mediated by the binding of SHP to NF-κB [149].

Macrophages express both FXR and TGR5. Macrophages from FXR-knockout mice exhibit enhanced release of pro-inflammatory cytokines [147]. CDCA and FXR agonists reduce IL-6 expression in macrophages. However, IL-6 expression is unaffected by FXR agonists in FXR-knockout mice [150]. Activation of TGR5 leads to the suppression of pro-inflammatory cytokines from LSP-stimulated macrophages [151]. The activation of macrophage TGR5 by INT-777, a TGR5 agonist, inhibits pro-inflammatory cytokine production. This inhibition is mediated by cAMP signaling, followed by NF-κB inhibition [151]. Furthermore, TGR5 activation promotes macrophage polarization from M1 (pro-inflammatory phenotype) to M2 (anti-inflammatory phenotype) macrophages and reduces pro-inflammatory cytokines [152,153,154]. In contrast, macrophage differentiation is unaffected by FXR-knockout [150].

Dendritic cells are antigen-presenting cells that play an essential role in the adaptive immune system and express TGR5. DCA suppresses lipopolysaccharide (LPS)-induced expression of pro-inflammatory genes, including IL-1, IL-6, and TNFα in dendritic cells. Furthermore, TGR5-knockout mice exhibit a recovery of LPS-induced expression of pro-inflammatory genes. These inhibitory effects of TGR5 are mediated by the repression of NF-κB via TGR5–cAMP–PKA signaling [155].

Recent studies have shown that 3-oxoLCA and isoalloLCA regulate T cell differentiation. IsoalloLCA triggers oxidative phosphorylation in mitochondria, leading to the production of reactive oxygen species (ROS). ROS from the mitochondria, not from the cytoplasm, induces forkhead box P3 (FOXP3). FOXP3 is a transcription factor and a master regulator of the development of regulatory T cells (Tregs), which release immunosuppressive factors. This 3-oxoLCA acts as an inverse agonist of retinoid-related orphan receptor γt (RORγt), leading to the inhibition of the development of T helper 17 (Th17) cells, which release pro-inflammatory factors. Both 3-oxoLCA and isoalloLCA are not produced in germ-free mice [156,157]. It has been reported that *Odoribacteraceae* strains can produce isoalloLCA via 5α-reductase and 3β-HSDH. Moreover, isoalloLCA shows selective antibacterial activity against gram-positive multidrug-resistant pathogens [56].

### 3.3. The Role of Modified BAs in the Nervous System

Both conjugated and unconjugated BAs can be detected in human and rodent brains [7,8,158,159]. Although the origin of BAs in the brain has not been confirmed, BAs can be transported from the peripheral circulation by BA transporters or diffuse across the blood–brain barrier (BBB) [4,160]. As CA, CDCA, and DCA are lipophilic [161], these unconjugated BAs can pass through the BBB via passive diffusion. There is a correlation between the brain and serum levels of CA, CDCA, and DCA [162,163]. On the other hand, conjugated BAs are large molecules that are negatively charged at physiological pH, so these BAs rely on transporters to pass through the BBB [164,165]. Furthermore, BAs increase BBB permeability through the phosphorylation of occludin, which is involved in the formation of tight junctions in the BBB [166,167]. Thus, the brain communicates with the gut and gut microbiome through BAs. This is known as the microbiome–gut–brain axis or the gut–brain axis. N-methyl-D-aspartate (NMDA) receptors in the brain are crucial for learning and memory because they induce long-term potentiation and long-term depression [168,169,170]. In contrast to NMDA, γ-aminobutyric acid type A (GABA_A_) receptor activation inhibits neurotransmission [171,172]. CA, CDCA, and DCA hinder the activation of NMDA and GABA_A_ receptors [173]. UDCA increases arousal by inhibiting GABA_A_ receptor activation in neurons in the tuberomammillary nucleus of the hypothalamus [174]. G/T DCA and DCA activate M2 and M3 muscarinic acetylcholine receptors, respectively, and these receptors play an important role in cognitive function, memory, and learning [175,176,177]. Furthermore, tauroursodeoxycholic acid (TUDCA) induces neural stem cell proliferation and neural differentiation in the subventricular zone of the lateral ventricles in rats [178]. Additionally, the neuroprotective role of TUDCA, a taurine-conjugated secondary BA, has been demonstrated in models of neurodegenerative diseases, including Alzheimer’s, Parkinson’s, and Huntington’s diseases [179].

MRGPRX4, also known as sensory neuron-specific G-Protein coupled receptor 5/6, is involved in cholestatic itch [62,180,181]. DCA, CDCA, CA, and LCA evoke itching of human skin via the activation of MRGPRX4 expression in dorsal root ganglion (DRG) neurons [62]. Furthermore, DCA, TDCA, UDCA, and CDCA evoke scratching behavior in mice expressing human MRGPRX4 [63].

## 4. Concluding Remarks

BAs are synthesized in the liver as primary BAs, and a variety of secondary BAs are synthesized in the intestine through the involvement of various microorganisms. These BAs bind to multiple nuclear and cell-surface receptors. Therefore, BAs have been recognized as signaling molecules. Each BA has a different affinity for the receptor to which it can bind. The abundance ratio of primary and secondary BAs may affect short- and long-term health. Thus, factors such as food, aging, and antibiotics affect the gut microbiome and may change the BA composition and affect health. Currently, probiotics, prebiotics, synbiotics, and postbiotics are considered substances that maintain health by preventing metabolic diseases by controlling the composition of the gut microbiome [182]. A recent study demonstrated that centenarians have a unique gut microbiome that produces iso-, 3-oxo-, allo-, 3-oxoallo-, and isoalloLCA [56]. Further elucidation of the role of the gut microbiome and the modification of BAs by the gut microbiome will lead to the development of new strategies for treating disease and improving human health.
